# Mucosal neuroimmune mechanisms in gastro-oesophageal reflux disease (GORD) pathogenesis

**DOI:** 10.1007/s00535-023-02065-9

**Published:** 2024-01-14

**Authors:** Tom Leech, Madusha Peiris

**Affiliations:** Centre for Neuroscience, Surgery and Trauma, Blizard Institute, Faculty of Medicine and Dentistry, Queen Mary University of London, 4 Newark Street, London, E1 2AT UK

**Keywords:** Gastro-oesophageal reflux disease, Neuroimmune, Mucosal immunity

## Abstract

Gastro-oesophageal reflux disease (GORD) is a chronic condition characterised by visceral pain in the distal oesophagus. The current first-line treatment for GORD is proton pump inhibitors (PPIs), however, PPIs are ineffective in a large cohort of patients and long-term use may have adverse effects. Emerging evidence suggests that nerve fibre number and location are likely to play interrelated roles in nociception in the oesophagus of GORD patients. Simultaneously, alterations in cells of the oesophageal mucosa, namely epithelial cells, mast cells, dendritic cells, and T lymphocytes, have been a focus of GORD research for several years. The oesophagus of GORD patients exhibits both macro- and micro-inflammation as a response to chronic acidic reflux at the epithelium. In other conditions of the GI tract, such as IBS and IBD, well-characterised bidirectional processes between immune cells and mucosal nerve fibres contribute to pathogenesis and symptom generation. Sensory alterations in these conditions such as nerve fibre outgrowth and hypersensitivity can be driven by inflammatory processes, which promote visceral pain signalling. This review will examine what is currently known of the molecular pathways linking inflammation and sensory perception leading to the development of GORD symptoms and explore potentially relevant mechanisms in other GI regions which may indicate new areas in GORD research.

## Introduction

Gastro-oesophageal reflux disease (GORD) is a condition that affects approximately 20–25% of UK adults, and its prevalence continues to increase globally [1]. The primary symptom of GORD is heartburn, described as the sensation of pain or burning in the distal oesophagus or epigastric region [2]. Heartburn is a type of visceral pain, resulting from the activation of afferent sensory nerve fibres in the oesophageal mucosa. Activation of oesophageal sensory afferents is likely to result from several factors, including impaired barrier function, inflammation or microinflammation, and afferent sensitisation [3–6]. These factors indicate communication between noxious refluxate, epithelial cells, innate and adaptive immune systems, and mucosal sensory nerves.

GORD patients suffer chronic, long-term pain symptoms, which arises partly from the sensitisation of sensory nerves, both peripherally and centrally [7]. Innervation of the oesophagus is provided by spinal and vagal nerve branches that relay action potentials generated in afferent nerve fibres of the oesophageal mucosa to the central nervous system (CNS) [8–10]. In the vagal pathway, signals are transmitted to the nucleus tractus solitarius in the brainstem via nodose and jugular ganglia [8]. In the spinal pathway, afferent nerves synapse with cell bodies in the thoracic and cervical dorsal root ganglia (DRG) [8]. Sensory innervation to the oesophageal mucosa is provided by both vagal and spinal pathways, as shown by calcitonin gene-related peptide (CGRP) immunostaining in afferent fibres [11]. Spinal afferents are considered critical in nociception in response to acid reflux and sensitisation due to inflammation [10]. The molecular mechanisms underlying this sensitisation are beginning to be elucidated, with neuro-immune mechanisms likely to contribute significantly, similar to other visceral pain conditions [12–15].

In many peripheral organs such as the lungs, skin, and proximal and distal gastrointestinal (GI) tract, characterised neuroimmune interactions, communication between cells of the nervous and immune systems, are known to play a role in pain perception in various pathologies [12]. In the GI tract, these processes contribute to homeostatic and pathological functions [16–18]. In irritable bowel syndrome (IBS), a condition marked by visceral pain and microinflammation of the distal GI tract, several neuroimmune pathways are well defined, including sensitisation of nociceptive neurones by immune mediators such as histamine [13]. In inflammatory bowel disease (IBD), a chronic inflammatory condition of the large intestine, similar neuroimmune networks have been identified that promote visceral pain, as well as inflammation through interactions between neuropeptides and immune cells [14, 15]. Visceral pain signalling in the lower gut is mediated primarily via extrinsic networks, specifically sensory nociceptive neurones with cell bodies in the DRG innervating all layers of the lower GI tract [19]. Therefore, insights from lower GI tract pathologies are suggestive of potential oesophageal neuroimmune mechanisms relevant to GORD.

**Fig. 1:**
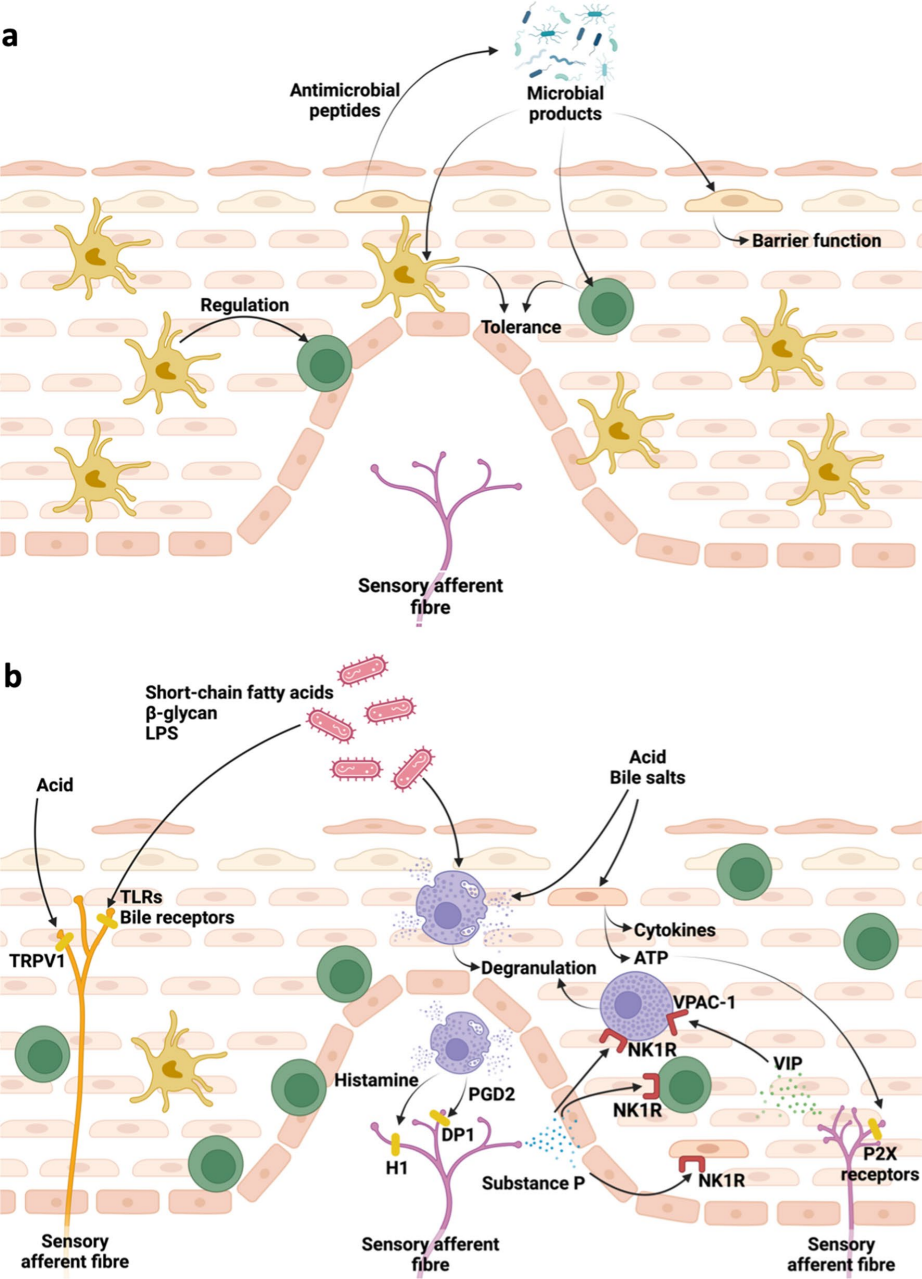
Potential neuro-immune interactions in the oesophagus of GORD patients, resulting in pain and inflammation. **a** In the healthy oesophagus, microbial products, epithelial cells, and mucosal immune cells may act symbiotically to promote mucosal tolerance and barrier function. **b** In GORD oesophagus, bacterial dysbiosis may lead to immune cell activation and direct activation of sensory afferent fibres. High levels of reflux as well as increased epithelial permeability allow exposure of superficial sensory nerves, immune cells, and epithelial cells to acid and bile salts, resulting in the production of inflammatory mediators, as well as TRPV1 activation of CGRP + sensory nerves. Degranulating mast cells release histamine and prostaglandins, including prostaglandin D2 (PGD2) which may activate H1 receptor and Prostaglandin D2 receptor 1 (DP1) on sensory afferent nerve fibres, respectively. Substance P released from afferent fibres in response to neuronal activation may bind NK1R on mast cells, T lymphocytes, and epithelial cells to promote the expression of inflammatory genes. Created with BioRender.com

There is a wealth of evidence describing chronic inflammation in the oesophageal mucosa of GORD patients, regardless of macroscopic inflammation. Emerging evidence also points to the role of alterations in mucosal sensory innervation in GORD, specifically in driving heartburn symptoms. The aim of this review is to summarise current evidence of neuroimmune interactions in the oesophageal mucosa in GORD, as well as draw parallels with other GI mucosal barrier sites, both proximal and distal, which have established evidence of neuroimmune interactions in pathology. Possible neuroimmune interactions in GORD are summarised in Fig. 1.

## Neuroanatomy of the oesophagus

Both vagal and spinal afferents are likely to be important in pain signalling and extra-oesophageal symptoms such as anxiety and depression in GORD [20]. The anatomical location of these afferent fibres in the distal oesophagus, as well as their mucosal pattern could prescribe a role in the sensing of refluxed stomach contents.

Spinal nerves with ganglia in the spinal cord and which signal to the CNS, are implicated in causing, for example, heartburn [21]. Spinal afferent innervation of the oesophagus is provided via the DRG [8]. In the oesophagus of the cat and dog, retrograde tracing of DRG projections in the oesophagus has demonstrated spinal afferents localised at all levels of the oesophagus [22, 23]. CGRP has been used as a marker for spinal sensory afferents in the oesophagus due to its abundance in nerve fibres originating from the DRG and its paucity in vagal nerve fibres, as has been confirmed in mammals through combined retrograde labelling and immunohistochemistry [24, 25]. In the rat, CGRP + spinal nerve fibres are present throughout the length of the oesophagus, with many projections terminating in the mucosa [26]. In humans, CGRP + nerve fibres have been detected in all layers of the oesophagus, including the lamina propria and epithelium, throughout the length of the oesophagus [4, 5, 27]. These mucosal spinal afferents are likely involved in pain processing, and in the context of GORD, their proximity to the lumen could allow for the detection of noxious contents.

The oesophagus receives vagal innervation from C-fibres originating from nodose and jugular ganglia [28]. The vagus nerve contains sensory and motor nerve fibres with functions including gastro-oesophageal junction (GOJ) function and mechanosensation in the smooth muscle layer of the human oesophagus [29]. Calretinin, a neuronal calcium-binding protein, has been used as a putative immunohistochemical marker for vagal afferents in the oesophagus as it is present in retrograde labelled afferent neurones originating from the nodose and jugular ganglia but not the DRG [25]. Calretinin + nerve fibres have been found to be densely expressed in all layers of the upper and lower third of the rat oesophagus [25]. In this study, DRG neurones did not positively stain for calretinin, suggesting a high specificity for vagal neurones [25]. In mouse oesophagus, AAV-GFP labelling of vagal fibres from jugular/nodose ganglia revealed vagal innervation throughout the oesophageal mucosa [30]. In guinea pig, both jugular and nodose vagal afferents have been found throughout the length of the oesophagus, although their localisation within the mucosa has not been thoroughly investigated [28]. In humans, neurones containing neuropeptide Y (NPY) and vasoactive intestinal peptide (VIP), which are found in vagal nodose fibres, have been detected in the oesophageal mucosa [31–33]. These vagal afferents within the oesophageal mucosa have the potential for chemosensation or detection of inflammatory mediators due to their proximity to the epithelium.

Classically, vagal afferents have been thought to be involved in sensing physiological stimuli, However, evidence points towards the activation of peripheral afferent vagal fibres in the control of mood and pain modulation, as well as peripherally released neuropeptides from vagal afferents and efferents in immune homeostasis [34, 35]. Studies in guinea pig oesophagus show vagal afferents, specifically jugular C fibres, are activated by acid via TRPV1 and can also respond to pathological distension, suggesting a role in nociception [36]. Although a population of oesophageal vagal afferents have nociceptive properties, knowledge of contribution to pain perception is limited. Current evidence suggests vagal afferents may have a role in pain signal modulation [35], likely via projections from the nucleus tractus solitarius (NTS) which is involved in central pain processing [37]. The role of vagal activation in visceral pain is only partially understood, however, signalling through vagal afferents may also contribute to affective-emotional disorder in visceral pain conditions [20]. Vagal afferent activation also appears to produce an analgesic effect in some conditions. For example, high intensity electrical activation (≥ 150 μA) of capsaicin-sensitive vagal afferents in rats inhibits spinal nociception in response to thermal and mechanical stimuli at the skin, likely via descending vagal pathways [38].

Understanding the nociceptive capabilities of vagal afferents and alterations in GORD could reveal a role for this pathway in pain perception and affective symptoms. In an immunohistochemical study of the human oesophagus, percentage of papillae containing VIP + nerve fibres was increased in inflamed mucosa compared to healthy controls [32]. Further, in an ex vivo study, almost all guinea pig oesophageal vagal nodose neurones were found to be responsive to bradykinin via B2R [39]. Bradykinin is released by cell types including epithelial cells and immune cells in response to acid exposure [40, 41]. Therefore, some vagal afferents in the oesophageal mucosa have chemosensory ability and may be involved in pain modulation in response to noxious stimuli in GORD.

## Neurochemistry of the oesophagus in GORD

### CGRP

As described previously, CGRP is a key neuropeptide in nociception in the GI tract due to it being localised mainly to spinal nerve fibres originating from the DRG [19]. For this reason, CGRP is often used to characterise sensory neurones likely to be involved in pain perception. CGRP acts as a neurotransmitter in the DRG via a G-protein coupled receptor (GPCR) containing the protein subunit RAMP1 [42]. In rat oesophagus, CGRP is expressed in almost all neurones (99%) originating from levels C2-T12 of the DRG, but only a small proportion (2%) of vagal nodose and jugular neurones innervating the distal oesophagus [24, 26]. Therefore, neuronal CGRP reactivity in the distal oesophagus, where heartburn symptoms of GORD originate, is found predominantly in spinal afferent fibres. Activation of these spinal afferents by luminal contents such as refluxate or by inflammatory products results in the perception of pain, for example, oesophageal perfusion of pH ~ 1 pepsin solution in rats results in potent activation of CGRP + nerves in the DRG [11, 43]. In healthy human distal oesophageal mucosa, CGRP + nerve fibres are expressed in the lamina propria as well as some projections into the epithelium [11]. However, in the oesophageal mucosa of NERD patients, CGRP + nerve fibres are significantly more dense compared to healthy volunteers, and this CGRP + nerve fibre density was negatively correlated with oesophageal distention pain threshold [44]. However, in an immunohistochemical study of ERD oesophageal mucosa, no difference was found in the percentage of papillae which contained a CGRP + nerve fibre [32], a result that may be explained by the criteria used by this study that excluded the epithelium and subepithelial lamina propria. Increased density of CGRP + sensory nerve fibres may be important in heartburn symptoms specifically in NERD patients.

Notably, CGRP-containing nerve fibres lay closer to the luminal surface of the oesophageal epithelium in NERD patients compared to healthy controls [4]. This difference is not seen in functional heartburn (FH), ERD, or Barrett’s Oesophagus (BE) [4, 45]. In NERD oesophagus, a significantly greater proportion of epithelial CGRP + nerve fibres are positive for the proton receptor, TRPV1, compared to ERD patients [5]. Taken together, these CGRP + TRPV1 + nerve fibres are likely to be involved in sensing acidic refluxate and the perception of pain in GORD. NERD patients characteristically retain an intact epithelial barrier compared to ERD, and therefore the superficiality of sensory afferents could be critical in their sensing of noxious luminal contents compared to ERD, which is typified by epithelial erosions [46].

Given current evidence, it is unlikely that CGRP has direct effects on epithelial and immune cells in the oesophageal mucosa. However, it is probable that the alterations in the location and receptor expression of CGRP + nerve fibres in NERD augments nociception, likely partly via immune interactions. For example, NGF secreted by intestinal mast cells can contribute to nerve growth under stress conditions, resulting in hypersensitivity [47, 48]. Furthermore, increased sensitisation of TRPV1 on CGRP + neurones in GORD could potentially contribute to nociception. This may be attributed to inflammatory or bacterial products, including ATP, and TLR agonists such as HMGB1 and LPS, which has been demonstrated in DRG neurones in vitro and in vivo [49]. Endogenous mediators of TRPV1 sensitisation, such as ATP may contribute to neuronal hypersensitivity in GORD [40]. In a rat model of oesophagitis, mRNA expression of ATP receptor P2X3 in vagal and spinal afferents was higher compared to controls [50]. Further research is required to determine the expression of immune product receptors on sensory afferents in the human GORD oesophagus, as this could elucidate mechanisms of neuronal sensitisation leading to heartburn.

### Substance P

Substance P (SP) is a neuropeptide contained in a subtype of nociceptive neurones and is found in both spinal and vagal nerves. In the oesophagus of mammals, including the opossum and the cat, SP plays a role in oesophageal peristalsis and the function of the lower oesophageal sphincter (LES), where it is co-released with acetylcholine (ACh) by vagal efferent nerves [24, 51, 52]. In pain signalling of the spinal pathway, SP released from afferent sensory nerve fibres binds preferentially to neurokinin 1 receptor (NK1R) within the dorsal horn [53]. In the opossum oesophagus, SP-containing nerve fibres are present in all layers of the oesophagus, including the mucosa [54, 55]. SP + nerve fibres are present in healthy human oesophageal mucosa [31] and the percentage of papillae containing SP + neurones have been observed to be unchanged between healthy controls and ERD patients [32]. However, the mucosal protein level of SP is elevated in NERD patients [56], with a negative correlation between SP + neurone density and oesophageal distention pain threshold [44].

**Fig. 2:**
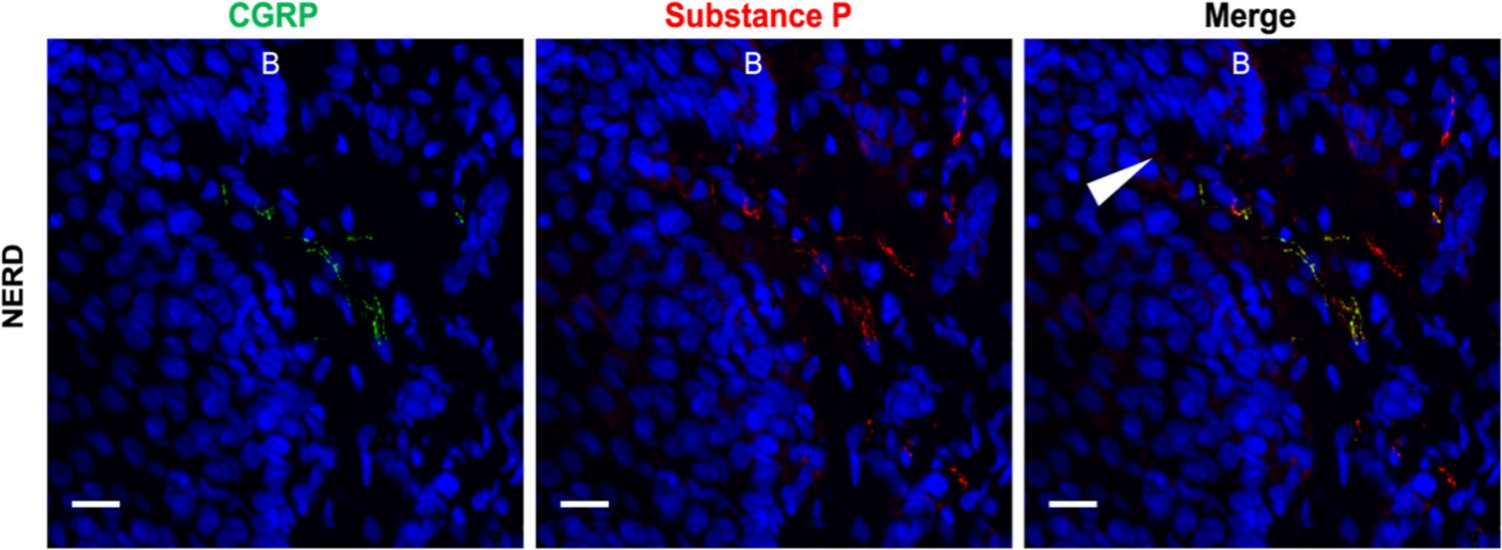
CGRP + Substance P + nerve fibres in a papilla of a non-erosive reflux disease (NERD) patient. The basal layer of the epithelium is indicated with a ‘B’. The papilla is marked with an arrowhead. Scale bar = 20 µm

More research is needed to determine alterations in SP innervation in GORD phenotypes, as this may contribute to visceral hypersensitivity. However, it’s possible that elevated levels of sensory SP + neurones play a role in pain perception in NERD and not ERD as SP-ergic innervation appears to be altered specifically in NERD oesophagus. As SP is found in both vagal and spinal fibres, activation of SP + afferents could contribute to visceral pain as well as anxiety/depression symptoms in GORD. An image of a CGRP + SP + (spinal) nerve fibre in the oesophageal mucosa is shown in Fig. 2.

### Neurogenic inflammation in GORD

Neurogenic inflammation is a conserved process, occurring in the skin, trachea, bladder, and GI tract [57, 58]. Sensory afferent nerve fibres at barrier sites participate in neurogenic inflammation which requires sensing of noxious components such as acid (via TRPV1), bacterial/viral components (via TLRs), or ATP released from damaged epithelial cells (via P2X receptors) [59]. Subsequent neuronal activation results in rapid local release of neuropeptides such as SP and CGRP from afferent endings which act on cell types including epithelial, endothelial, and leukocytes, invoking a rapid inflammatory response [19, 53, 59]. SP binding to NK1R on endothelial cells leads to reduced endothelial integrity and upregulation of leukocyte adhesion molecules, facilitating plasma and leukocyte extravasation [58, 60]. SP + neurone density is altered in gut pathologies including in IBS patients where density in colonic mucosa is threefold higher compared to healthy controls [61] with similar findings in IBS subtypes [62, 63]. High SP + nerve fibre density is also reported in inflammatory skin conditions prurigo nodularis, psoriasis, and atopic dermatitis [64].

There is emerging evidence of SP-mediated neurogenic inflammation in the oesophagus. An ex vivo study of cat oesophageal tissue showed pH2 incubation-induced neuronal SP release via TRPV1 [65]. Interestingly, NK1R mRNA has been reported to be increased in both NERD and ERD mucosa [56, 66]. This is consistent with inflammatory conditions of the skin with a neurogenic inflammatory component, such as chronic prurigo [67]

CGRP, a potent vasodilator at local blood vessels [68], promotes leukocyte infiltration to the injury site during acute inflammation but also acts as an anti-inflammatory mediator [14, 69]. In the skin, local release of CGRP upon neuronal activation contributes to oedema and leukocyte infiltration [70]. However, CGRP may also have an anti-inflammatory function in the skin and on endothelial cells by downregulation of NF-κB activation [71]. Indeed, CGRP knockout mice with DSS-induced colitis suffer worse symptoms driven by decreased activation of TGF-β-expressing CD4 + Tim4 + intestinal macrophages [72]. This data suggests CGRP may have an anti-inflammatory role at barrier sites, particularly during long-term high-grade inflammatory conditions such as IBD, but its function as a vasodilator facilitates acute inflammation. RAMP1 mRNA expression is low in the oesophageal mucosa of healthy individuals and is not altered in NERD, suggesting mucosal CGRP does not act locally [56].

## Neuro-immune crosstalk in GORD

It is widely accepted that GORD is likely a condition driven by mucosal inflammation rather than direct acid damage [73]. Several cell types, comprising epithelial, immune, and neuronal cells are involved in the inflammatory pathogenesis of GORD. In vitro studies have indicated that the components of refluxate, acid and bile salts, induce the production of inflammatory mediators such as COX-2, IL-1β, and IL-8 in oesophageal epithelial cell lines [74, 75]. In patients with ERD and NERD, there is chronic upregulation of inflammatory cytokines including IL-1β, IL-8, IL-33, TNF-⍺, and IFN-γ [74, 76–78]. Among the immune cells implicated in GORD pathogenesis and likely to release the aforementioned cytokines, are mast cells, eosinophils, dendritic cells, and T/B lymphocytes.

### Mast cells

Mast cells have been a focus of lower GI pathology research for many years, and have the most evidence supporting a neuro-immune function. In NERD patients, there are a significantly higher number of mast cells in oesophageal mucosa compared to healthy volunteers, as well as a greater proportion of degranulated mast cells [79]. Zhong et al*.* reported a greater number of intraepithelial mast cells in patients with reflux chest pain syndromes (RCS), which includes patients with retrosternal and epigastric pain as main symptoms, rather than typical reflux symptoms such as regurgitation [80]. Therefore, oesophageal mast cell degranulation may contribute to heartburn symptoms in GORD patients. However, it is necessary to be cautious when comparing immune cell counts between oesophageal biopsies due to inherent limitations. For example, as immune cells are present in greater numbers in the lamina propria compared to the epithelium, the proportion of the biopsy containing these two layers can drive alterations in mucosal immune cell counts if not controlled for. Other limitations include sloughing off of the most luminal epithelial layers during biopsy preparation, which can inflate cell counts. Finally, endoscopic oesophageal biopsies typically contain almost entirely epithelium which, among other factors, can make the biopsy difficult to orientate and papillae difficult to visualise when counting intrapapillary immune cells.

In IBS, a relationship between mast cells and sensory nerves has been described [81]. One study found the number of mast cells within 5 μm of a nerve fibre to be greatly increased in the mucosa of the descending colon in IBS patients compared to healthy controls, as well as a higher proportion of degranulated mast cells in the descending colon mucosa [82]. The number of mucosal mast cells within 5 μm of a nerve fibre, determined with electron microscopy, was correlated with the severity and frequency of abdominal pain [82]. In eosinophilic oesophagitis (EoE), a high density of mucosal mast cells within the oesophagus is associated with heartburn perception, and the mast cells are in close proximity to TRPV1 + nerve fibres [83]. Although, this pattern of proximity is not as clear as in the lower gut, as mast cells are not as close to nerve fibres.

Mast cell products can sensitise neurones within the GI tract, for example, in guinea pig oesophagus, mast cell-derived histamine and prostaglandin D2 have been found to sensitise vagal nodose C-fibres to distension via H1 and DP1 receptors, respectively [84, 85]. Activation of these receptors increases firing of guinea pig airway vagal afferents, partly due to sensitisation of TRPV1 via intracellular GPCR signalling cascades [86, 87]. In IBS, the degranulation state of mast cells, rather than just density, is important in symptom generation [88]. More research is required to investigate the degranulation states of mast cells in GORD and downstream impacts of mast cell products, including histamine and NGF, on mucosal nociceptors.

Mast cells may contribute to neuronal hypersensitivity in GORD, but neuropeptides released in neurogenic inflammation may also, in turn, promote mast cell degranulation, driving symptom generation. Cultured human mast cells express receptors for SP and VIP, and incubation of mast cells with SP or VIP can lead to degranulation [89]. The number of mast cells expressing VPAC-1, a VIP receptor, was found to be increased in EoE [90]. Precise interactions between oesophageal VIP + neurones and mast cells have not been elucidated but it is possible that increased vagal activation in response to reflux could promote mast cell degranulation. Research is required to determine the nature of neurone-mast cell signalling in GORD. A likely interaction, which requires further study, may be SP signalling via NK1R to promote mast cell degranulation, driving neuronal hypersensitivity and epithelial permeability. The possible functions of mast cells in GORD are summarised in Fig. 1.

### Eosinophils

Eosinophils are also innate immune cells involved in inflammatory diseases of the GI tract. Epithelial eosinophils are very rare in healthy controls, as well as a very low mRNA level of eosinophil chemotaxins such as eotaxin1-3 and eosinophil maturation mediators such as IL-5 [91–93]. As the main diagnostic criterion for EoE is ≥ 15 eosinophils/ HPF, epithelial eosinophil infiltration is expectedly increased in these patients [94]. Epithelial mediators released during allergen challenge induce a type 2 inflammatory response, leading to the production of eosinophil chemotactic and maturation mediators including eotaxin1-3, which are upregulated in EoE [93]. Pathological functions of infiltrating eosinophils include barrier disruption by granule proteins such as major basic protein (MBP), mast cell recruitment and activation by IL-9, as well as tissue remodelling by IL-13 [95]. Eosinophils have also been shown to express neurotrophic factors such as NGF as well as various mediators that may promote neuronal activation such as MBP [96]. However, EoE therapeutics, including monoclonal antibodies, targeting eosinophils have been unsuccessful in improving clinical outcomes, suggesting that eosinophils may be symptomatic, rather than drivers, of disease progression in EoE [95, 96].

Most studies investigating epithelial eosinophil infiltration in GORD have described a low number of intraepithelial eosinophils, particularly in comparison with EoE [91, 92, 97–99]. However, there is a moderate increase in mucosal mRNA levels of eotaxin1-3 between healthy controls and GORD patients [93]. Also, one study applying a lower threshold of eosinophil infiltration (≥ 5 eosinophils/ HPF) found eosinophil infiltration in 26.1% of GORD patients with oesophagitis and 35.7% of GORD patients without oesophagitis studied, compared to 0% of healthy controls [92]. Eosinophil infiltration in the oesophagus may be related to dysphagia symptoms [100]. GORD patients listing dysphagia as their primary symptom display a significantly greater number of eosinophils/HPF compared to those listing dysphagia as a secondary or tertiary symptoms, as well as patients without dysphagia [100]. This could be related to fibrosis induced by eosinophil-derived mediators. Overall, there is disagreement surrounding the extent of eosinophil infiltration in GORD, which could be prescribed to different pathologists, counting, and biopsy collection methods as described earlier. Although eosinophils may be an important factor in some GORD symptoms such as dysphagia, they are unlikely to be an effective treatment target in this disorder.

### Dendritic cells

Dendritic cells (DCs) are antigen-presenting cells (APCs) that link innate and adaptive immune systems. DCs in the oesophagus are not well characterised during homeostatic or pathological conditions. In Barrett’s oesophagus, a condition defined by intestinal metaplasia of the distal oesophagus due in part to chronic acid reflux, CD83 + DCs are present in the lamina propria and form clusters with T and B lymphocytes [101]. DCs of the human intestine are phenotyped based on expression of protein markers, primarily the presence of CD103 [102]. CD103 expression defines the conventional DC1 subset, which has a role in cross-presentation of exogenous antigens to lymphocytes [102]. Similar categorisations exist for DCs in blood as well as at other barrier sites such as the gastric mucosa and skin [103]. Although there is a limited characterisation of oesophageal DCs when compared to those of the intestine, there is evidence of a CD103 + CD11c + DC population in healthy oesophageal mucosa. However, most oesophageal DC research relates specifically to adenocarcinoma and tumour infiltrating DCs.

In contrast to the intestinal mucosa, it is known that Langerhans cells (LC), a subset of immature DCs that express CD1a, are commonly found in the oesophageal epithelium, in particular the suprabasal region [104, 105]. LCs are powerful antigen-presenting cells (APCs) and, therefore, play a role in maintaining antigenic tolerance or promoting immunity at barrier sites [106]. In the skin, LC function has been characterised. During steady state, when no exogenous antigen is detected, LCs promote the proliferation of CD4 + Tregs, dependent on contact-mediated interactions via MHC class II and CD80-86, as well as the release of the cytokines IL-2 and IL-15, leading to peripheral tolerance [106–108]. LCs also prime antigen-specific T-lymphocytes during an inflammatory response [109]. The microenvironment of the epidermis is critical in driving LC function. Indeed, TGF-β, a mediator released by keratinocytes during homeostatic conditions is important in inhibiting LC maturity and their retention in the epidermis [110]. Whereas, intradermal injection of TNF-α, which is released by epidermal keratinocytes during inflammation, promotes LC migration from the epidermis to the dermis [111]. LCs in the oesophagus are likely to respond in similar ways to the epithelial microenvironment, which may contribute to the inflammatory epithelial T cell response which is observed early in GORD onset.

Due to the paucity of studies regarding DC quantification and localisation in the oesophagus, it remains difficult to theorise potential neuroimmune interactions between DCs and afferent nerve fibres in the oesophagus. However, in the human colonic mucosa, CD103 + (“conventional DC1”) DCs were found to be in close apposition to CGRP + sensory nerve fibres [112]. No such relationship has been demonstrated in the oesophagus. In murine-derived DCs, NK1R agonism in vitro increases expression of co-stimulatory and activation markers CD80, CD83, CD86, CD40, and MHC class II [113]. NK1R agonism also inhibits DC production of IL-10, but not IL-1β, IL-6, or TNF-⍺. In vitro challenging of murine CD11c + DCs with SP provokes NF-κB activation [114]. Whereas, in vitro treatment of cultured LC-like cells with VIP reduces their capacity to present antigen to T cells whilst downregulating IL-12 and IL-1β production [115]. Therefore, there could be bi-directional communication via sensory pathways which may be altered in GORD.

### Lymphocytes

T lymphocytes have a demonstrated role in the onset of GORD, and their activity is regulated by interactions with tissue DCs. In an oesophagoduodenostomy model of reflux oesophagitis, T-cell infiltration into the oesophageal mucosa is an initial event preceding the development of oesophagitis [116]. Similarly, in GORD patients, 1 week after discontinuing PPI treatment, intraepithelial lymphocyte infiltration is significantly increased [117]. A study by Osman et al*.* showed epithelial T lymphocyte counts are reduced following 1 month of PPI treatment [118]. Taken together, these studies suggest a rapid and reversible increase in mucosal lymphocyte infiltration in response to acid reflux. This finding has been consistently noted in cross-sectional studies, describing an T lymphocyte epithelial infiltration in GORD patients in various patient cohorts [80, 92, 119, 120]. An in vitro study by Huo et al*.* showed condition media from the oesophageal epithelial cell line NES-B10T exposed to acidic bile salt promoted T cell migration [75]. Therefore, soluble mediators released from epithelial cells in response to reflux may be responsible for driving T-cell infiltration into the epithelium. The exact function of these infiltrating T lymphocytes, however, as prescribed by the method of their activation and subsequent molecular expression, remains largely unknown.

The most comprehensive study of T cell subtypes in GORD investigated cell surface marker expression of ex vivo cultured T cells from healthy control, Barrett’s Oesophagus, and reflux oesophagitis patients [121]. T lymphocytes isolated from reflux oesophagitis patients displayed an inflammatory phenotype, with a high proportion of cytotoxic Granzyme B + cells [121]. Two cross-sectional studies found intraepithelial T lymphocytes to be predominantly CD8 + in the healthy and inflamed oesophagus [120, 122]. A study of 200 reflux oesophagitis patients found the ratio of CD4 + :CD8 + T cells to be approximately 2:1, which is within the healthy range for adults [118, 123]. However, the focus of this study on circulating T cells rather than T cells in the oesophageal mucosa severely limits its application in mucosal pathogenesis. Lastly, a study of paediatric patients found an increased proportion of Treg cells (FOXP3 +) in the oesophageal mucosa in EoE and GORD [119]. However, due to the young age of recruited patients, this may not be directly translatable to adult GORD, as paediatric and adult GORD can differ in clinical presentation, and T cell populations alter dramatically with age [124, 125]. Overall, T cells appear to be involved in the pathogenesis of oesophagitis in GORD. The inflammatory microenvironment of the GORD oesophagus may be responsible for promoting T cell maintenance and altered gene expression. Further characterisation of T cell populations in GORD is required, including NERD and FH, the pathogenesis of which is still unclear.

**Fig. 3:**
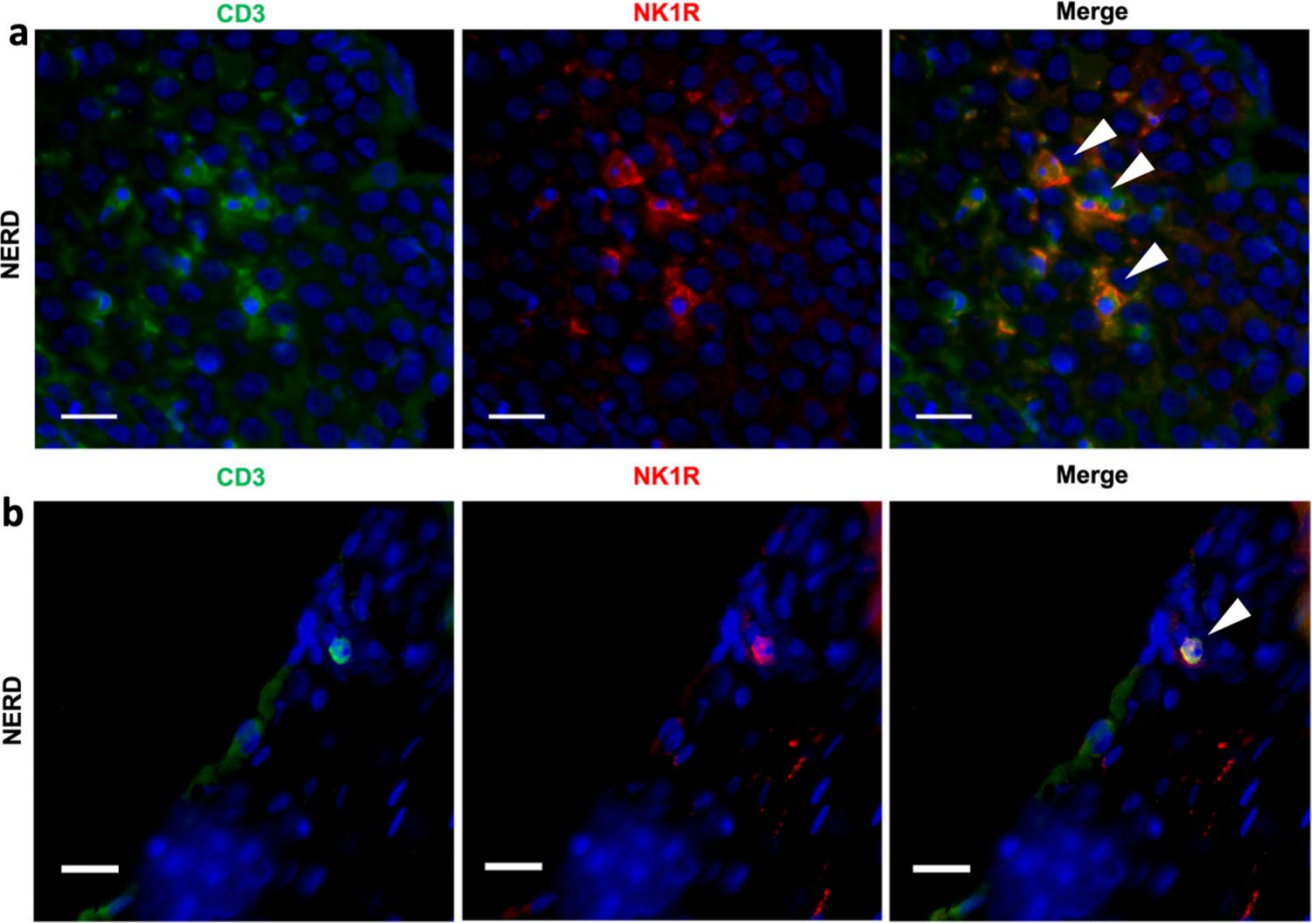
NK1R + T lymphocytes in the oesophageal epithelium of two non-erosive reflux disease (NERD) patients. **A** Interepithelial T lymphocytes positive for the substance P receptor, NK1R. **B** A T lymphocyte in an oesophageal papilla expressing NK1R. NK1R + T lymphocytes are marked with an arrowhead. Scale bar = 20 µm

In vitro studies have established a role of SP in T cell activity. We have demonstrated the expression of NK1R on a population of T lymphocytes in the oesophageal mucosa of GORD patients, as shown in Fig. 3. NK1R expression on T cells can be induced by the mucosal microenvironment. For example, in a model of NSAID-induced colitis, NK1R expression was induced in T lymphocytes of IL-10 knockout animals [126]. Further in vitro experimentation revealed that Th1 cytokine IL-12 promoted and IL-10 inhibited NK1R expression in isolated mouse CD4 + T cells [126]. IL-12 also promoted NK1R expression in a human T cell line [127], possibly via an NF-κB-mediated pathway [128]. NK1R activation on human T lymphocytes induces expression of macrophage inflammatory protein-1β (MIP-1β), enhancing T cell chemotaxis [129]. Additionally, NK1R agonism may play a role in augmenting T cell cytokine production, as in T cells isolated from IL-10 knockout mice with NSAID-induced colitis, TGF-β and SP were required for upregulated production of IL-17 and IFN-γ [130]. Therefore, SP may specifically augment migration, activation, and subsequent cytokine release of mucosal T cells in GORD patients. NK1R expression on T cells appears highly dependent on the microenvironment and more research is required to determine how this may affect NK1R + T cells in GORD.

Unlike T lymphocytes, B lymphocytes are not well researched in the oesophagus. In contrast to the intestinal wall, the oesophagus does not contain Peyer’s patches, organised lymphoid follicles containing clusters of T and B cells. However, B cells have been observed in the papillae and lamina propria, but not the epithelium, of the healthy oesophagus [131]. Epithelial and papillary CD20 + B cell infiltration is higher in EoE compared to healthy controls [131, 132]. However, EoE is an allergic-mediated disease and is associated with other Mendelian atopic conditions [133]. In EoE, exposure of the epithelium to allergens induces the release of Th2-priming mediators including IL-33 [133]. These Th2 cells then secrete IL-13, promoting B cell chemotaxis and proliferation [133]. Therefore, EoE pathogenesis is markedly different from GORD. Indeed, epithelial B lymphocyte infiltration during GORD pathogenesis has been shown to be minimal [117]. Although, one study has described a high number (700 ± 30/10 HPFs) of clustered B cells in the oesophageal mucosa of GORD patients which is markedly reduced (10 ± 2/10 HPFs) by 1 month of PPI treatment [118]. However, these figures and observations are outliers and can possibly be attributed to the aforementioned issues of cell counting from oesophageal biopsies, particularly the depth of the biopsies.

### The oesophageal microbiome and its potential role in neuroimmune pathways

The gut microbiome has received increasing attention as a regulator of mucosal inflammation, permeability, and neuronal sensitivity [134, 135]. Throughout the intestine, commensal bacterial products interact directly with cells of the immune system, including DCs, T lymphocytes, and epithelial cells to promote tolerance and maintain epithelial function [134]. These interactions are facilitated by microbial products, including short-chain fatty acids (SCFAs) and polysaccharides, released by intestinal flora which resides in the mucus layer [134]. Antimicrobial peptides including β-defensins and secretory leukocyte protease inhibitor (SLPI) produced by epithelial cells limit host exposure to commensal bacteria [134]. The particular species which make up the microbiome vary greatly through the length of the GI tract, and depend on the luminal environment, including pH and nutrient levels, however, 90% of bacterial species in healthy human faecal samples are of *Firmicutes* and *Bacteroidetes* phyla [136, 137].

Disruption in the GI microbiome has been implicated in the pathogenesis and progression of several conditions including infection, metabolic syndromes, IBD, and IBS [134]. As well as its role in epithelial permeability and inflammation, accumulating evidence suggests that alterations in the microbiome is also involved in the onset of pain symptoms in GI conditions [135]. Mice treated with antibiotics (bacitracin/neomycin) for 10 days caused hypersensitivity to colorectal distension compared to placebo-treated mice [138], with a higher concentration of myeloperoxidase in tissue and higher SP innervation in the submucosa [138], both of which were normalised following co-administration of live *Lactobacillus paracasei* and its metabolic products [138]. In a study of zymosan-induced colitis in neonate rats, supplementation with *Lactobacillus rhamnosus GG* for 39 days rescued hypersensitivity to colonic distension [139], coinciding with altered levels of 5-HT, noradrenaline, and dopamine in various CNS regions [139]. This suggests the role of the microbiome in regulating hypersensitivity to painful stimuli, potentially involving central sensitisation. Commensal bacteria may also exert anti-nociceptive functions directly on sensory neurones, as *Lactobacillus reuteri* inhibited TRPV1-mediated activation of DRG neurones [140]. Other bacterial products such as LPS, β-glycan, or SCFAs may also bind directly to receptors on peripheral neurones, including TLRs and bile acid receptors, to induce peripheral sensitisation of sensory neurones [135]. Therefore, dysbiosis in the microbiome of the GI tract can result in bacteria acting directly, or indirectly via immune cells, on afferent neurones to induce or protect from hyperalgesia. Possible impacts of the oesophageal microbiome and bacterial dysbiosis in GORD pathogenesis are summarised in Fig. 1.

The oesophagus supports a distinct resident microbiome [141, 142], and lacks a permanent mucous layer, allowing bacteria to directly adhere to squamous cells [141, 143]. The majority of bacteria populating the distal oesophagus are of *Firmicutes* (70%) followed by *Bacteroidetes* (20%) phylum [141]. Similar bacterial proportions have been reported using 16S rRNA analysis of oesophageal biopsies as well as oesophageal secretions collected by a string test [144–146].

Several studies have found altered oesophageal microbiomes in oesophageal disease patients [146–149]. Unsupervised clustering analysis of 16S rDNA from distal oesophageal biopsies of healthy volunteers and ERD patients revealed two clusters based on the combined genetic distance of microbiome samples [148]. The ‘Type I’ microbiome contained predominantly *Firmicutes* phylum, specifically *Streptococcus* genus, with 11/12 healthy oesophageal samples belonging to this group [148]. However, 7/12 of oesophagitis samples had a ‘Type II’ microbiome, characterised by a lower proportion of *Streptococcus* genus and a higher proportion of gram-negative anaerobes/microaerophiles [148]. Bacterial load in the distal oesophagus is higher in GORD patients compared to healthy controls, with no change in number of taxa present (α-diversity) between groups [146]. GORD patients treated with PPIs had a lower proportion of *Firmicutes* and a greater proportion of *Proteobacteria* bacteria compared to healthy patients, perhaps as a result of low-acid reflux altering the oesophageal microenvironment [146], although paediatric and adult samples were analysed together; age is a key variable in oesophageal microbiome composition [146, 149]. In a study by Deshpande et al.*,* α-diversity was unchanged between healthy controls and GORD patients [149]. However, gram-negative bacteria were present in greater numbers in GORD oesophagus and pathway analysis revealed differences in microbial pathways including lactic acid production, hexitol degradation, and heme biosynthesis [149]. Microbial dysbiosis could result in the production of products that act directly on afferent sensory nerves or alter the epithelial-immune homeostasis, resulting in the production of inflammatory mediators which sensitise or activate these nerves.

## Conclusion

Neuroimmune pathways are key mediators of both physiological and pathological processes. Several cell types are involved in these processes, including spinal and vagal afferent neurones, epithelial cells, bacteria, and immune cells. Visceral pain and chronic inflammation are major symptoms of GORD, and there are similarities between the cell types involved in other GI conditions. Emerging evidence describes sensory changes in the oesophageal mucosa of GORD patients in the presence of macro- and micro-inflammation. As described in the lower gut and other organs, these sensory alterations are likely to be driven partly by inflammatory processes, which has subsequent effects promoting visceral pain. Investigation of these interactions in the oesophagus could elucidate pathways leading to heartburn, particularly in NERD and FH patients. Further research is required to determine the exact mechanisms which are relevant in the oesophagus and develop future treatments which meet the needs of PPI-refractory GORD patients.
